# Efficient processing of natural scenes in visual cortex

**DOI:** 10.3389/fncel.2022.1006703

**Published:** 2022-12-05

**Authors:** Tiberiu Tesileanu, Eugenio Piasini, Vijay Balasubramanian

**Affiliations:** ^1^Center for Computational Neuroscience, Flatiron Institute, New York, NY, United States; ^2^Scuola Internazionale Superiore di Studi Avanzati (SISSA), Trieste, Italy; ^3^Department of Physics and Astronomy, David Rittenhouse Laboratory, University of Pennsylvania, Philadelphia, PA, United States; ^4^Santa Fe Institute, Santa Fe, NM, United States

**Keywords:** natural scene analysis, visual cortex (VC), textures analysis, efficient coding hypothesis, sensory system

## Abstract

Neural circuits in the periphery of the visual, auditory, and olfactory systems are believed to use limited resources efficiently to represent sensory information by adapting to the statistical structure of the natural environment. This “efficient coding” principle has been used to explain many aspects of early visual circuits including the distribution of photoreceptors, the mosaic geometry and center-surround structure of retinal receptive fields, the excess OFF pathways relative to ON pathways, saccade statistics, and the structure of simple cell receptive fields in V1. We know less about the extent to which such adaptations may occur in deeper areas of cortex beyond V1. We thus review recent developments showing that the perception of visual textures, which depends on processing in V2 and beyond in mammals, is adapted in rats and humans to the multi-point statistics of luminance in natural scenes. These results suggest that central circuits in the visual brain are adapted for seeing key aspects of natural scenes. We conclude by discussing how adaptation to natural temporal statistics may aid in learning and representing visual objects, and propose two challenges for the future: (1) explaining the distribution of shape sensitivity in the ventral visual stream from the statistics of object shape in natural images, and (2) explaining cell types of the vertebrate retina in terms of feature detectors that are adapted to the spatio-temporal structures of natural stimuli. We also discuss how new methods based on machine learning may complement the normative, principles-based approach to theoretical neuroscience.

## 1. Introduction: Sensory adaptation to natural environments

The sensory systems of animals face the challenge of using limited resources to process very large and high dimensional sensory spaces. For example, the olfactory systems of most animals use a few hundred to a thousand receptor types (Vosshall et al., 2000; Zozulya et al., 2001; Zhang and Firestein, 2002) to encode a vast number of mixtures of odorants drawn from the tens of thousands of possible volatile molecules (Dunkel et al., 2009; Touhara and Vosshall, 2009; Mayhew et al., 2022) with corresponding challenges for encoding and decoding odors (see Singh et al., 2021; Krishnamurthy et al., 2022) and references therein). The visual system faces the similarly acute problem of encoding the relevant information in continuously changing scenes composed of photons with frequencies that range continuously across the visual spectrum, light intensities spanning over 10 orders of magnitude (Tkačik et al., 2011), and a vast diversity of possible textures, shapes, objects, and kinds of motion. Overall, the challenge is that the visual input is extremely high dimensional and the human retina must manage the formidable task of encoding it with just three receptor types (red, green, and blue cones) during daytime, processed *via* the retinal network into about 20 types of visual feature detectors (retinal ganglion cells) that tile the visual space (Balasubramanian and Sterling, 2009).

The challenge of performing difficult tasks with limited resources and many constraints recurs across all the scales and domains of life. There is also a standard tactic used by living things to mitigate this challenge: evolutionary adaptation of systems to the environment or to the tasks required by the environmental niche, a notion that goes back to Darwin and his observation of the finches of the Galapagos (Darwin, 1859). Applied to sensory systems, this tactic requires adaptation of sensory algorithms, circuit architectures, and cell properties to the statistical structure of the environment on multiple timescales. Broadly, this suggests that the fixed architecture of sensory systems should be adapted over evolutionary times to the typical statistical structure of the environment, while plasticity supports fine tuning to the detailed differences that distinguish specific environments during the lifetime of individuals.

One powerful formulation of this idea is the *efficient coding hypothesis*. Different authors have adopted somewhat different formulations of this hypothesis, but we will take it to state that neural systems commit their limited resources to maximize the information relevant for behavior that they encode from the environment. To formulate this principle precisely we must define what we mean by “information,” “relevant,” and “behavior”. However, in the sensory periphery, a standard approach is to simply assume that neural circuits do select behaviorally useful data from the environment (e.g., bright vs. dark local contrast extracted by retinal ON and OFF cells), and to ask instead how the circuits should be structured, and how computational resources should be allocated, to maximize the encoded information given biological constraints such as the number of available cells or the amount of ATP that the circuit can consume (see, e.g., Atick and Redlich, 1990; Ratliff et al., 2010 in retina, Teşileanu et al., 2019 in the olfactory epithelium, and Wei et al., 2015 in the entorhinal cortex). In information theoretic terms these approaches ask how peripheral sensory circuits should be organized to maximize the mutual information of their outputs with the environment, given constraints of noise, ATP consumption (Attwell and Laughlin, 2001; Balasubramanian, 2021; Levy and Calvert, 2021), the number of available cells, and the like.

Thinking in this way, researchers have explained many aspects of early vision, e.g., nonlinearities in the fly visual system (Laughlin, 1981), center-surround receptive fields of neurons in the vertebrate retina (Atick and Redlich, 1990; van Hateren, 1992b; Vincent and Baddeley, 2003; Kuang et al., 2012; Pitkow and Meister, 2012; Simmons et al., 2013; Gupta et al., 2022), spike timing statistics (Fairhall et al., 2001), the preponderance of OFF cells over ON cells (Ratliff et al., 2010; Gjorgjieva et al., 2014), the mosaic organization of ganglion cells (Borghuis et al., 2008; Liu et al., 2009), the scarcity of blue cones and the large variability in numbers of red and green cones in humans (Garrigan et al., 2010), selection of predictive information by ganglion cells (Palmer et al., 2015; Salisbury and Palmer, 2016), and the expression of ion channels in insect photoreceptors (Weckström and Laughlin, 1995). Similar analyses suggest that the auditory (Schwartz and Simoncelli, 2001; Lewicki, 2002; Smith and Lewicki, 2006; Carlson et al., 2012) and olfactory (Teşileanu et al., 2019; Singh et al., 2021; Krishnamurthy et al., 2022) peripheries are also adapted to the statistical structure of the environment so that they use limited resources efficiently to represent sensory information (Sterling and Laughlin, 2015). While many of these analyses have focused on linear filtering properties, some have focused on the nonlinear separation of the visual stream into separate information channels like bright and dark spots or color channels (Garrigan et al., 2010; Ratliff et al., 2010; Gjorgjieva et al., 2014). It remains a challenge for the future to understand the complete repertoire of nonlinear visual features extracted by retinal ganglion cells (Gollisch and Meister, 2010) in these terms, a task that will likely require an extension of previous methods to include the temporal dynamics of natural scenes, the computational complexity of the required decoding network, and the mutual information between aspects of the visual stimulus and important behaviors.

Similar principles may also apply more centrally in the thalamus and in primary visual cortex, for example in asymmetries between ON and OFF responses (Komban et al., 2014; Kremkow et al., 2014) and the structure of receptive fields (Olshausen and Field, 1996; Bell and Sejnowski, 1997; van Hateren and van der Schaaf, 1998; Vinje and Gallant, 2000). In auditory cortex, contrast gain control has been shown to facilitate information transmission and help detecting signals against a noisy background (Rabinowitz et al., 2011; Angeloni et al., 2021). There is even evidence that the grid system in the entorhinal cortex acts as an efficient encoder of space (Wei et al., 2015). In this article we follow this line of thinking further, and describe recent studies that show that deeper layers of visual cortex in multiple species are adapted to the spatial and temporal statistics of natural scenes. In Section 2, we discuss the representation of visual textures in cortex, which occurs in areas V2 and above in humans. In Section 3, we discuss experiments showing that the findings in humans also extend to rodents. In Section 4, we discuss a case where behavioral relevance is more narrowly defined, namely the representation of object identity in the visual ventral stream, and how representations that are invariant to identity-preserving transformations (e.g., changes in viewpoint) can be learnt from the temporal statistics of natural scenes. We conclude in Section 5 with a discussion of challenges for the future.

## 2. Adaptation to spatial statistics and texture perception

Above, we have discussed how the requirement of maximizing information transmission shapes the earliest visual layers. At the level of the retina and primary visual cortex (V1), neurons are often presumed to be mostly sensitive to simple, first- or second-order image statistics (e.g., Atick and Redlich, 1990; van Hateren, 1992b; Olshausen and Field, 1996; Borghuis et al., 2008; Pitkow and Meister, 2012; Simmons et al., 2013), although responses to complex spatio-temporal features are evidently also present in the phenomena like motion anticipation (Berry et al., 1999), lag-normalization (Trenholm et al., 2013), the omitted stimulus response (Schwartz et al., 2007), and nonlinear feature detection (Gollisch and Meister, 2010). As sensory data is further processed through the visual hierarchy, neurons develop selectivity for more complex visual elements such as shapes (Pasupathy and Connor, 1999; DiCarlo and Cox, 2007; Rust and DiCarlo, 2010) and textures (Landy and Graham, 2004; Freeman et al., 2013; Okazawa et al., 2015). Are these neurons are also tuned to maximize the efficient transfer of information? To approach this question, we first need a precise definition of what a visual texture is.

Intuitively, textures are images with “distinctive local features [...] arranged in a spatially extended fashion” (Victor et al., 2017); at a formal level, however, textures are best defined in terms of *ensembles* of images (Victor, 1994; Portilla and Simoncelli, 2000; Victor et al., 2017) because multiple different images can represent the same texture. Indeed, the statistical regularities of a texture patch, and not the precise spatial arrangement of light intensity, yield its perceptual quality. By grouping all images that are perceived as a single texture type into a statistical ensemble, we are effectively describing the statistical properties of the image that are important for defining that type of texture. However, despite the ensemble nature of textures, a single image is typically sufficient to identify a texture. It must thus be possible to infer statistical properties related to the whole ensemble from a single patch, provided the patch is large enough. This suggests that texture ensembles possess the property of *ergodicity*: spatial averaging coincides with ensemble averaging (Victor, 1994; Portilla and Simoncelli, 2000; Victor et al., 2017).

Within this framework, a specific type of texture is defined by a set of constraints on image statistics. This could be, for instance, fixing the average luminance, or the correlation between luminance values a certain distance apart. In general infinitely many such constraints may be needed to fully specify a texture ensemble, but a maximum-entropy approach is often used to pick out a unique ensemble that fixes only a finite number of statistics (Portilla and Simoncelli, 2000; Zhu et al., 2000; Victor et al., 2017). The entire set of textures that can be obtained by varying the values of a chosen class of image statistics can be arranged in a *texture space*, with each axis representing a statistic. These axes are generally not independent: they are subject to inter-dependencies and mutual constraints (Victor et al., 2017).

Several alternative texture parameterizations have been used in the literature (e.g., Victor and Conte, 1991; Portilla and Simoncelli, 2000; Zhu et al., 2000; Tkačik et al., 2010; Hermundstad et al., 2014; Teşileanu et al., 2020). Here we focus mainly on grayscale textures with a finite number of discrete luminance levels, and multi-point correlations restricted to small (2 × 2 or 3 × 3) neighborhoods (Victor and Conte, 1991; Tkačik et al., 2010; Hermundstad et al., 2014; Teşileanu et al., 2020).

To test efficient coding of such visual textures we need three ingredients. First, we need a method for analyzing natural images to measure the distribution of textures that is likely to be encountered by an animal. Second, we need a mathematical formalism for making testable predictions based on the natural distribution. And third, must create an experimental paradigm that allows us to test these predictions.

The first step is relatively straightforward given the formal definition of texture space described above. It amounts to selecting a dataset of natural images (e.g., van Hateren and van der Schaaf, 1998), splitting each image into patches assumed to have roughly constant texture^1^, and then for each patch calculating the image statistics that define the chosen texture space. Next, each texture patch can be characterized by its coordinates in texture space, and typical efficient-coding calculations can be used to estimate the properties of an optimal filter that maximizes the transmitted information (van Hateren, 1992a; Hermundstad et al., 2014). Notably, the scale-invariance of natural images (Field, 1987; Stephens et al., 2013) implies that the outcome of this first step will not be strongly dependent on the resolution of the images (i.e., the size of a pixel); indeed, this prediction has been partially tested by Hermundstad et al. (2014), who reported results that were consistent independently of the scale of the initial block-averaging operation in their image-processing pipeline.

A natural experimental approach for checking the predictions of efficient coding models is to measure the neural or behavioral responses of human or animal subjects to patches of known textures. The preferred method for obtaining texture patches is to generate them artificially, as this avoids biases introduced by contextual information that might be available in patches cropped from natural images (Julesz, 1962). This method also scales more easily to very large sample sizes, which are needed to analyze higher-order statistics. Texture generation can be time consuming, but a variety of powerful algorithms have been developed for the class of textures that we are considering here (Victor and Conte, 2012; Piasini, 2021).

**Figure 1:**
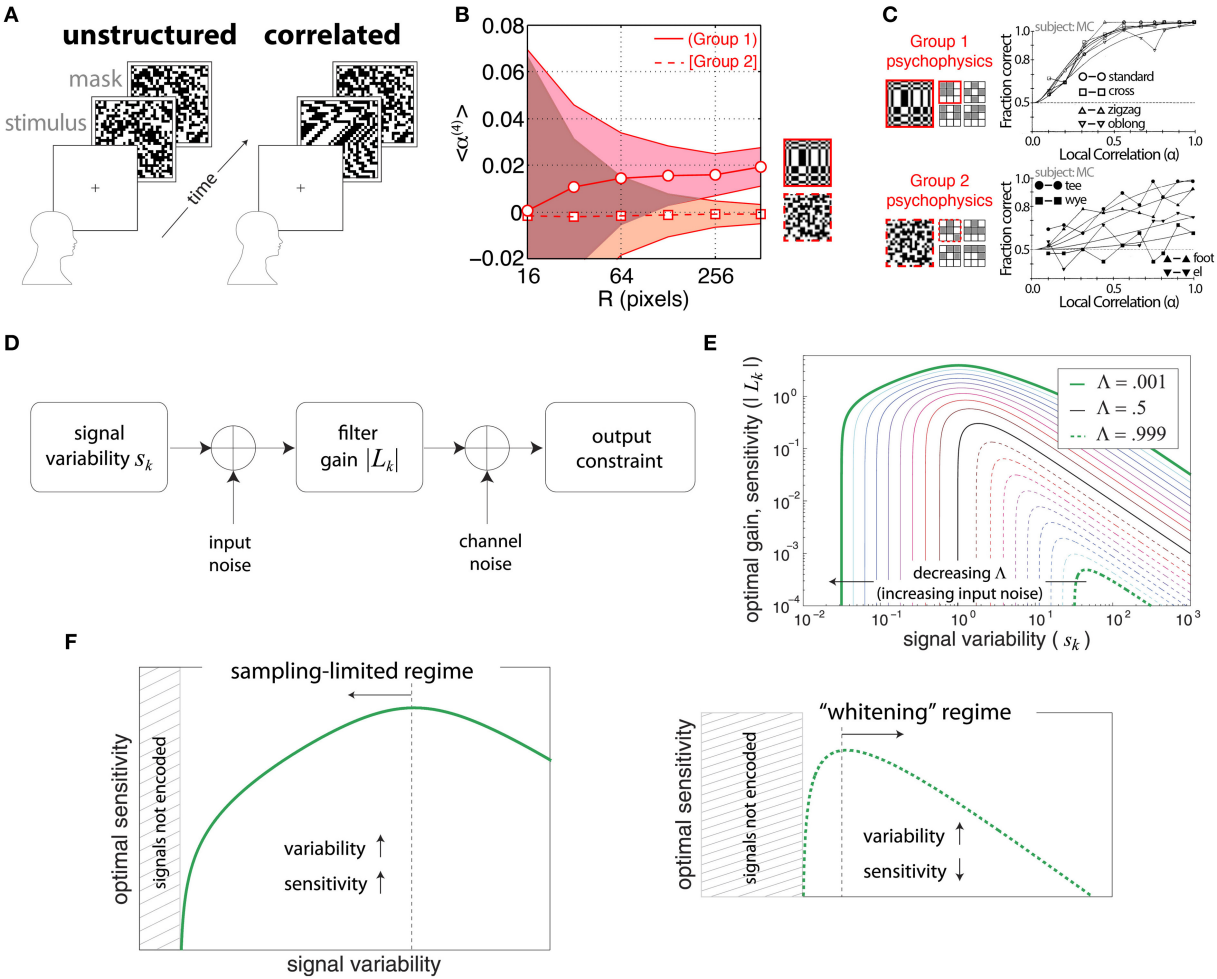
Efficient coding relating natural-image statistics to psychophysics. **(A)** The forced-choice task from Victor and Conte (1991). After a cue, a subject is shown either an unstructured (Victor and Conte, 1991) texture half of the time, or a correlated texture. The subject was asked to distinguish between the structured and unstructured stimuli. **(B)** Average four-point correlations calculated for each of two kinds of glider (see **C**) over a database of natural images. Group 1 correlations have an average that is statistically positive, while Group 2 correlations average close to zero even for large patches. Adapted from Tkačik et al. (2010). **(C)** Psychophysics results for the same texture groups as in **(B)**. The *x*-axis shows the strength of the correlations. Group 1 textures have low discrimination thresholds, while Group 2 textures are hard to distinguish from unstructured noise even at the highest correlation levels. Adapted from Victor and Conte (1991) and used with permission from Elsevier. **(D–F)** Two regimes of efficient coding, depending on input and output noise. Adapted from Hermundstad et al. (2014). **(D)** The model that is being optimized. **(E)** Results of the optimization as a function of input noise. Note that higher gain leads to higher sensitivity assuming a fixed threshold on the amplified signal. Signal variability is the variance of the input signal. The parameter Λ measures the balance between input and output noise. Small Λ is the regime where input nose dominates, while large Λ is when output noise dominates. **(F)** (Left) The “variance predicts salience,” sampling-limited regime that was found to be relevant for texture perception in Hermundstad et al. (2014). (Right) The more familiar whitening regime of efficient coding.

Victor and Conte (1991) used a forced-choice task to determine psychophysical thresholds for discriminating certain binary textures involving fourth-order correlations from unstructured textures (Figure 1A) with independent, identically distributed pixels. The fourth-order correlations were defined in terms of the relative positions of the four points involved in the correlation, shown pictorially as a *glider* in Figure 1C. Tkačik et al. (2010) used the same gliders to analyze correlations in natural scenes, drawn from a database of pictures taken in the African savannah (Tkačik et al., 2011). They found that the most informative^2^ natural-image correlations matched the texture dimensions that were more salient in the psychophysical trials. Conversely, the correlations that were least informative in the natural-image database were the ones that human subjects had difficulty distinguishing from unstructured noise (compare Figures 1B,C)^3^.

Note that we are concerned with the *correlations* between pixels with relative positions determined by a glider. For example, if we want to consider a four-point correlation in which the four pixels are arranged in a certain way we represent it as a glider as in Figure 1. But drawing a glider in this way does not imply that the pixels in the glider must all have the same luminance, or indeed any other particular pattern of brightness. For example, a vertical two-point correlation could be significantly different from 0 even if a pattern consisting of two bright pixels arranged on top of each other never occurred in the image patch. This can for instance happen in a patch of alternating bright and dark pixels, which would lead to a highly negative vertical two-point correlation.

A later analysis (Hermundstad et al., 2014) focused on just one of the gliders identified as salient in Victor and Conte (1991) and Tkačik et al. (2010), the 2 × 2 glider shown with a solid red outline in Figure 1C, along with correlations between pairs and triples of pixels within this glider. In this study, a comparison between natural-image statistics and psychophysics revealed that textures that exhibited higher variance in natural scenes were also more perceptually salient—in other words, “variance predicts salience” (Hermundstad et al., 2014). This effect can be explained as a result of the presence of significant input (sampling) noise compared to output noise (Hermundstad et al., 2014; Figures 1D–F). We can understand why this occurs in the simple example of linear efficient coding models. In this case, salience is related to the gain factor associated with each stimulus—a higher gain factor leads to smaller detection thresholds. When the main source of noise occurs at the output, amplification increases the signal-to-noise ratio. Then, if the total output power is fixed, the most efficient encoding whitens the signal—this is the scenario that has been studied most extensively. Hermundstad et al. (2014) noted, however, that when the *input* is corrupted by sampling noise, the signal-to-noise ratio can no longer be improved by amplification. In this regime, the best strategy is to de-emphasize the low-variance signals, which are corrupted by noise and thus not very informative, and instead use high gain factors for the high-variance, more informative signals. This highlights the importance of considering efficient-coding ideas that go beyond redundancy reduction by whitening.

**Figure 2:**
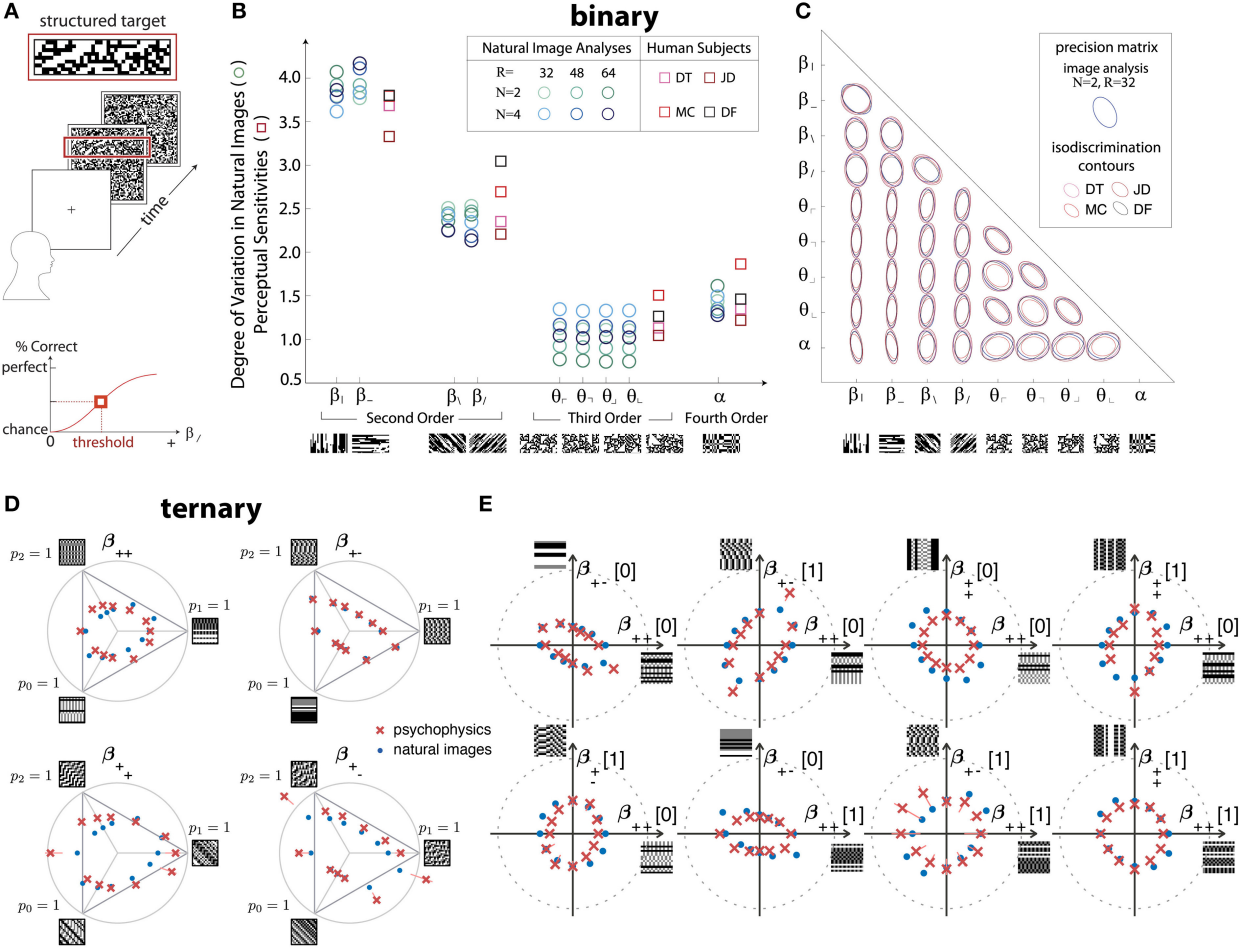
Efficient coding predicts detailed psychophysical thresholds for a variety of binary and ternary textures. **(A–C)** Results for binary textures, adapted from Hermundstad et al. (2014). **(A)** The four-alternative forced-choice (4AFC) design of the experiment: after a cue, subjects are shown a background of structured (unstructured) texture with a strip of unstructured (structured) texture in one of the four cardinal positions. The subjects need to identify the location of the strip. **(B)** Predicted (in various shades of blue) and measured (in shades of red and purple) perceptual sensitivities for various two-, three-, and four-point correlations. Sample patches are shown under the *x*-axis. The different shades correspond to different preprocessing choices (for the natural-image analysis) and different subjects (for the psychophysics). **(C)** Predicted (blue) and measured (shades of red and purple) isodiscrimination contours for textures that combine two of the 10 axes used in **(B)**. **(D,E)** Results for ternary textures, adapted from Teşileanu et al. (2020). **(D)** Predicted (blue) and measured (red) discrimination thresholds for textures in different “simple” planes of the grayscale texture space with three gray levels. See Teşileanu et al. (2020) for a detailed description of the texture space. **(E)** Predicted (blue) and measured (red) discrimination thresholds for textures in different “mixed” planes. See Teşileanu et al. (2020) for a detailed description of the texture space.

In greater detail, the work from Hermundstad et al. (2014) used a four-alternative forced-choice (4AFC) design to analyze the sensitivity of human subjects in multiple directions in the space of binary textures with up to four-point correlations contained within 2 × 2 blocks (Figure 2A). The relevant texture space was 10-dimensional (Victor and Conte, 2012), and the psychophysical trials assayed all of these dimensions (Figure 2B), as well as pairs of dimensions (Figure 2C). In all these cases, the variance of the correlations in natural images was an excellent predictor of the perceptual salience of the corresponding textures in psychophysical trials.

Going beyond binary luminance is challenging because, in this framework, the dimensionality of texture space for a four-pixel glider grows as the fourth power of the number of gray levels (Victor and Conte, 2012). In follow-up work, Teşileanu et al. (2020) introduced a new parameterization of the texture space for *G*>2 gray levels and focused on textures with three gray levels (ternary textures). Using the same psychophysics paradigm as Hermundstad et al. (2014), they probed more than 300 different rays in the resulting 66-dimensional texture space. Their results found an excellent match between most predicted and observed discrimination thresholds, in agreement with the earlier results for binary textures (Figures 2D,E).

Interestingly, the results from Teşileanu et al. (2020) also showed glimpses of limitations of the efficient coding idea: the prediction errors in a few texture-space directions were much larger than in the other directions (Figure 2D). This effect appears to be related to symmetries that act differently on natural-image ensembles as compared to human texture sensitivity. While the precise meaning behind the mismatches is not yet clear, general considerations suggest that we should expect deviations from the simplest forms of efficient coding because of resources limitations, alternative criteria that the brain might optimize or balance, etc. Of course, resource limitations can be incorporated into a more general information maximization problem *via* additional constraints, and other considerations like the utility of information for behavior can likewise be addressed. We return to these more general formulations of efficient coding principle in the Discussion.

**Figure 3:**
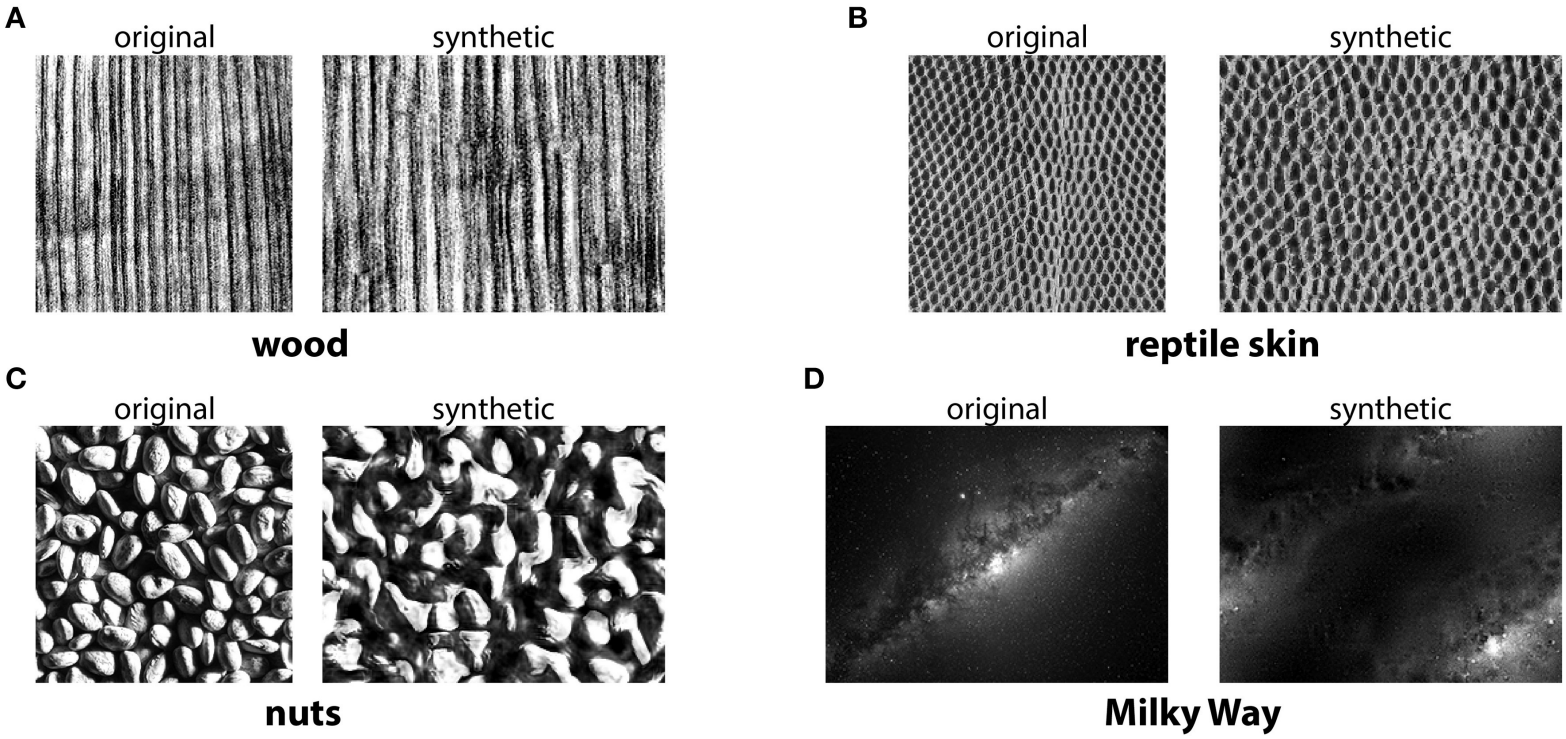
Photorealistic textures from the Portilla-Simoncelli texture model. The synthetic images were generated using the Matlab code from https://github.com/LabForComputationalVision/textureSynth. **(A–D)** The original samples were taken either from the same repository (for “reptile skin” and “nuts”), or from other freely available images on the internet (for “wood” and “Milky Way”).

The methods described above use a simplified texture space—a small number of gray levels, correlations constrained to small neighborhoods—in order to facilitate texture generation. Correspondingly, the resulting textures capture only a small fraction of the complexity of natural textures. A different approach is to exhaustively search for the statistics that are needed to generate a texture that is as indistinguishable as possible from its natural counterpart (Victor et al., 2017). Very powerful methods in this direction include the multiscale techniques proposed by Portilla and Simoncelli (2000) and recent approaches based on deep learning (Gatys et al., 2015; Ustyuzhaninov et al., 2016; Ding et al., 2020; Park et al., 2020). See Figure 3 for an example of photorealistic textures.

There are two fundamental downsides of more realistic texture models (Victor et al., 2017). First, texture generation can be significantly more time consuming—though perhaps this is less of an issue with modern hardware. Second, texture space can be much harder to describe and navigate: for instance, the model from Portilla and Simoncelli (2000) uses a parameterization in which many combinations of parameter values are not valid. This limitation can, however, be circumvented by reparameterizing the texture space in the vicinity of the manifold defined by naturally occurring textures (Lüdtke et al., 2015). Psychophysical studies using the textures from Portilla and Simoncelli (2000) suggest that peripheral vision is well-adapted to the distribution of such textures in natural scenes (Balas et al., 2009). A particularly striking illustration of this involves natural images that are modified using the texture model from Portilla and Simoncelli (2000) but look the same as the originals as long as the changes occur only in the visual periphery (Freeman and Simoncelli, 2011).

Yet a different approach is to use the symmetries of a specific texture model to generate new patches starting from texture patches cropped from natural images (Gerhard et al., 2013). If the model is accurate, the generated patches should be indistinguishable from natural textures as far as human observers are concerned. Conversely, if human subjects can distinguish between the natural and generated textures, the model must be incomplete. While all the models investigated by Gerhard et al. (2013) were incomplete in this sense, the discrimination performance was high for models that poorly fit natural-scene textures and low for models that provided a better fit. This suggests that the better models—the ones that yielded generated textures that were hardest to distinguish from natural textures—are the ones that are better adapted to natural-scene statistics, in agreement with efficient-coding ideas.

## 3. Extending to other animal species

In the previous sections we have described several experiments that illustrate how human vision, and in particular texture perception, is adapted to the statistics of natural visual scenes. Despite the power and flexibility afforded by human psychophysics, this approach also has limitations. For instance, investigation into the neural mechanisms for texture representation in humans rely on techniques such as fMRI (Beason-Held et al., 1998), as invasive recordings are not possible in humans. Developmental studies in humans also have very tight operational constraints (but see Gervain et al., 2021 for encouraging first steps), and of course causal manipulation of the developmental process is excluded.

In order to overcome these constraints, it is necessary to investigate perception and neural coding of visual textures in other animals.

Studies in macaque have investigated the encoding of multipoint correlations in visual cortex (Purpura et al., 1994; Yu et al., 2015), showing that representations of three- and four-point correlated patterns emerge prominently at the single cell level in V2 (Yu et al., 2015). However, dedicated psychophysical tests of the prediction of efficient coding theory are not available in these animals. In recent years, rodents have gained popularity as model systems for the study of vision. Rodents allow for running experiments with larger number of animals, and the anatomical layout of rodent visual cortex makes it easier to perform simultaneous recordings from distinct cortical areas contributing to the processing of visual stimuli (Glickfeld and Olsen, 2017; Tafazoli et al., 2017; Piasini et al., 2021). In particular, rats have been used successfully to investigate high-level processing involved in object recognition (Zoccolan et al., 2009; Tafazoli et al., 2017; Djurdjevic et al., 2018; Piasini et al., 2021). Moreover, unlike monkeys, rats are amenable to altered-rearing experiments, which were used to reveal how elementary coding features of primary visual cortex are adapted to the statistics of visual stimuli during development (Matteucci and Zoccolan, 2020). Overall then, rats are a convenient animal model for exploring efficient coding of visual textures.

**Figure 4:**
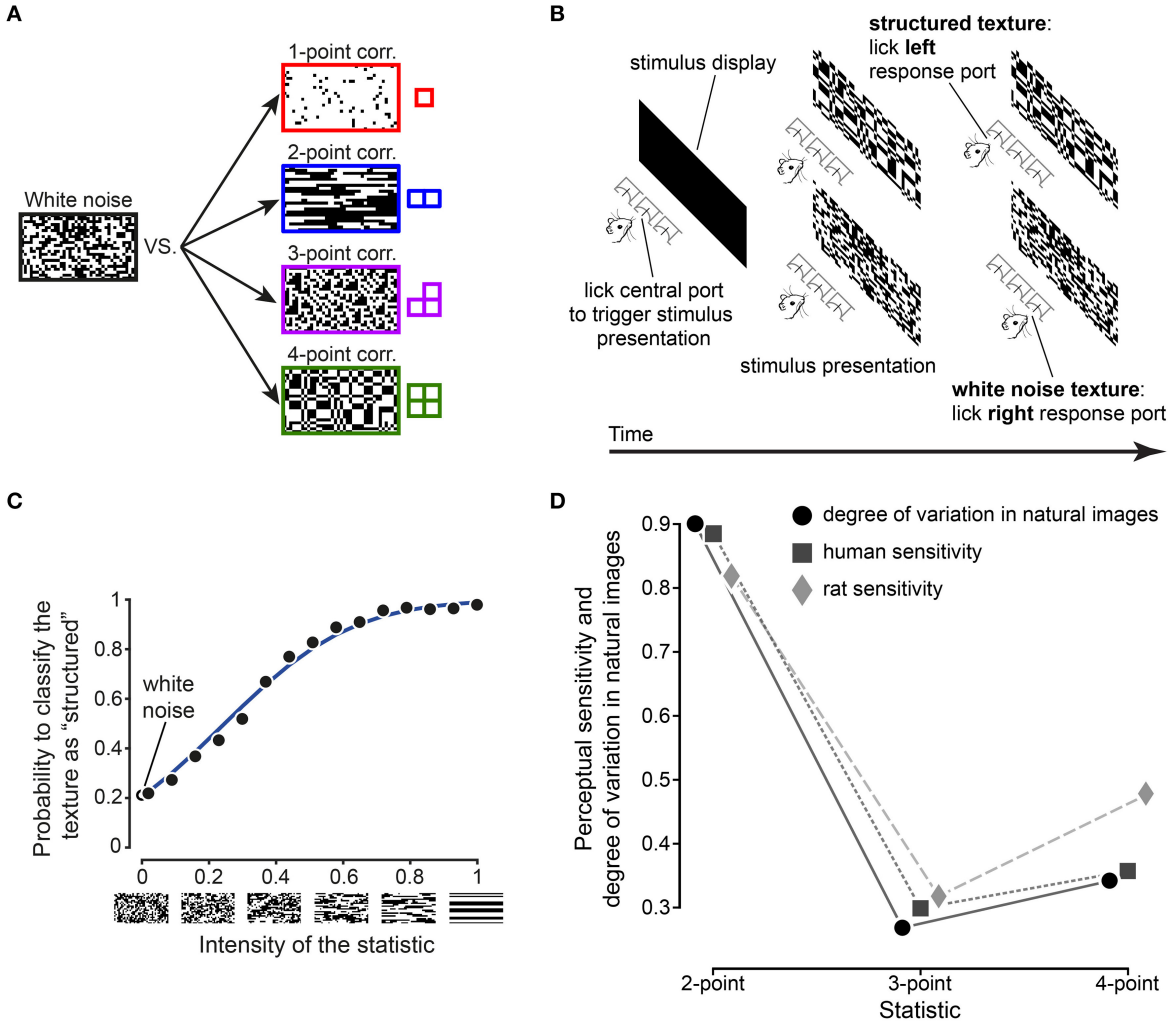
Rat sensitivity to visual multipoint correlations verifies the prediction from efficient coding theory, matching that found in humans. **(A)** Example stimuli used in the psychophysics task. Rats performed a two-alternative forced choice task where they had to report if a given stimulus was an instance of structured noise (a correlated pattern with one-, two-, three-, or four-point structure, generated using the gliders on the right), or unstructured “white” noise. **(B)** Operant structure of the psychophysics task. **(C)** Example psychometric curve from one of the rats trained to distinguish 2-point correlated textures from white noise. Black dots: experimental data. Blue line: ideal observer model fit. **(D)** Comparison between rat and human sensitivity to structured textures (diamonds and squares, respectively), and degree of variability of the corresponding statistics in natural images (dots). Adapted from Caramellino et al. (2021).

The first step in such an investigation is to establish whether rodents (and rats in particular) exhibit the same pattern of sensitivity to visual multipoint correlations as humans. In a recent study, Caramellino et al. (2021) designed a psychophysics task inspired by an experiment in humans (Victor and Conte, 2012; Hermundstad et al., 2014), and used it to probe rat sensitivity to visual textures. Briefly, rats were trained on a two-alternative forced choice task (2AFC), where a visual texture was presented on a monitor and the animal had to report if the texture was a sample of unstructured “white” noise (each pixel black or white with equal probability, independently from its neighbors), or if it was a sample from a maximum-entropy distribution with a nonzero level of one of four multipoint correlations (Figures 4A,B). A separate group of rats was trained for each type of correlation (one-, two-, three-, and four-point). The rats' behavioral performance was interpreted with the help of an ideal observer model (Figure 4C) tailored to the task design, which allowed estimation of the animal's perceptual sensitivity. This estimate matched the sensitivity measured in humans in the work described above (Hermundstad et al., 2014) as well as the degree of variability of multipoint correlations in natural images (Figure 4D). These results therefore show that texture perception in rats is adapted to the statistics of natural stimuli, in a way that closely matches both the prediction of efficient coding theory and analogous results in humans.

The results discussed here have opened the way to studying how higher-order visual properties are efficiently encoded in the brain. Rapid progress is now being made through the study of rodent vision. In a very recent preprint, Bolaños et al. (2022) present data linking texture perception and neural representation in mice, arguing that—compatibly with Yu et al. (2015) in macaque—useful representations of visual textures emerge in area LM (the rodent analog of V2). Importantly, the geometry of such representation seems predictive of the animals' behavioral performance in a visual discrimination task (texture vs. non-structured visual stimuli), and analogous results are reported for artificial neural networks. Together with Caramellino et al. (2021), this work is representative of a trend that uses texture perception as a tool to investigate vision in a broad set of species, including birds (Gervain et al., 2021) and even invertebrates (Reiter and Laurent, 2020).

## 4. Adaptation to temporal statistics and object recognition

The efficient coding principle as described above says that neural circuits are adapted to maximize the behaviorally relevant information they transmit, subject to appropriate constraints. While much of the research using this idea has focused on spatially organized information, adaptation to the temporal structure of stimuli as they arrive at the retina is also essential. This can occur on multiple timescales, from evolutionary adaptations to short-term sensory plasticity. In fact, some authors have proposed that long-range pairwise correlations in the luminance of natural scenes are not mainly removed in the retinal response by center-surround spatial receptive fields as usually presumed (Barlow et al., 1961; Atick and Redlich, 1990; van Hateren, 1992b; Barlow, 2001; Balasubramanian and Sterling, 2009), but rather by fixational eye movements and the resulting temporal effects on the retinal image (Kuang et al., 2012). More generally, the selection of information that is behaviorally relevant for an animal certainly involves temporal statistics, and may also involve state-dependent processes such as the influence of prior expectations, or feedback from the central brain. Indeed, already in the retina, the phenomena of motion anticipation and lag normalization (Berry et al., 1999; Trenholm et al., 2013) involve circuits that predict and use expected temporal regularities to adjust or enhance responses, and many of the nonlinear response features of retinal ganglion cells described in Gollisch and Meister (2010) also involve such effects. To ask whether these aspects of retinal coding can be understood in terms of efficient coding, we would ultimately need to identify how much utility they contribute to behavior, and not just how much information they convey. Perhaps the best-studied example of reformatting sensory information to make behaviorally-relevant quantities accessible occurs centrally, in the neural circuits of the visual cortex that represent object identity. Numerous theoretical works have proposed that useful object representations can be learned without supervision by adapting to statistical regularities of natural stimuli. We will now review these ideas and the associated challenges of accounting for state-dependent neural processing.

### 4.1. Unsupervised learning of invariant object representations

An important evolutionary advantage conferred by vision (Striedter and Northcutt, 2020) is the ability to detect and identify the objects in a visual scene. Accordingly, animals perform this task remarkably well by the standards of modern computer vision techniques. The visual ventral stream is a hierarchy of areas in the visual cortex of mammals that, to a first approximation, process object identity and encode aspects of the visual world in increasing order of abstraction—starting from simple features like edges in V1, to neurons in area IT (in primates) which are selective for object identity, and whose response is largely invariant to changes in location, point of view, illumination, and contrast (DiCarlo et al., 2012; Bao et al., 2020). More generally, such *transformation-invariant representations* have been recognized as a fundamental building block of an efficient object recognition system that can learn new categories from a limited number of examples (Anselmi and Poggio, 2014).

But how are these representations built by the brain? Behavioral (Cox et al., 2005) and electrophysiological (Li and DiCarlo, 2008; Matteucci and Zoccolan, 2020) evidence suggests that they are not hard-wired by evolution, but are at least partially the result of adaptation to the contingent spatio-temporal statistics of the natural world. Specifically, it has been proposed that the visual system learns to “discount” changes in size, pose, illumination etc. by exploiting the *temporal persistence* of objects encountered in visual scenes.

Indeed, the identity of the objects composing a scene typically possesses some degree of persistence from one moment to the next, while the low-level details of the impression those objects leave on a sensory system might change from moment to moment following changes in their position and configuration, or in the environment (e.g., illumination). Hence, to a first approximation, it may be reasonable for a sensory area to assume that the input patterns received from sensory transduction at two successive instants represent the same objects, modulo a set of transformations that do not affect object identity. This is the class of transformations that we wish neural representations to be invariant to, if the goal is to perform object recognition.

Different theories have been proposed for the mechanism of such adaptation. Földiák (1991) introduced a local synaptic learning rule which could lead to translation-invariant responses in a schematic model of complex cells in visual cortex. This line of inquiry was expanded in later years, leading to sophisticated models of representation learning across multiple areas of visual cortex (see for instance Wallis and Rolls, 1997; Masquelier et al., 2007). Another approach, at a higher abstraction level, is due to Wiskott and Sejnowski (2002), who introduced the Slow Feature Analysis (SFA) algorithm. For a data stream *X* = {*x*_0_, *x*_1_, …, *x*_*T*−1_, *x*_*T*_}, where $x_{t}\in {\mathbb{R}}^{n}$ is the content of the stream at time *t*, SFA finds a number of scalar features of the data *f*^*k*^ = *f*^*k*^(*x*) with maximal “slowness,” in the sense that (*df*^*k*^/*dt*)^2^ is minimized on average over the stream (under certain constraints that rule out trivial solutions). Notably, the features *f*^*k*^ are restricted to be instantaneous functions of the data—that is, $f_{t}^{k}=f^{k}\left(x_{t}\right)$, and do not depend explicitly on values of *x* at other times. The algorithm is therefore not allowed to resort to temporal filtering and must discover slowly-varying features that are computable from any “snapshot” of the data, for instance the identity of an object in a video stream that shows the object moving around in the visual field. SFA can be applied recursively to its own outputs, thus generating a representational hierarchy of increasing abstraction. Such a hierarchy could then form the backbone of a model of the visual system (Wiskott and Sejnowski, 2002; Körding et al., 2004; Einhäuser et al., 2005; Wyss et al., 2006; Franzius et al., 2007), or more generally of any object-recognition system, regardless of the sensory modality (DiTullio et al., 2022).

### 4.2. State-dependent processing

The models discussed above treat sensory information encoding as a feedforward cascade of stateless functions: at each point in time, the retinal input gets processed by a sequence of stages in a way that is independent of previous or later signals. In recent years, this approach to modeling the ventral visual stream has yielded impressive results, particularly by leveraging deep convolutional neural networks which allowed for accurate prediction (Yamins et al., 2014; Schrimpf et al., 2020) and even causal manipulation (Bashivan et al., 2019) of neuronal activity. Indeed, at least in rodents, convolutional neural networks have been shown to reformat visual information similarly to the ventral stream, according to notions of intrinsic dimensionality of population representations and of single cell-level distillation of elementary image features such as luminosity, contrast, edge orientation, and presence of corners (Muratore et al., 2022). However, phenomena such as short-term adaptation, or circuit features such as recurrent or feedback connectivity, can introduce state or history dependence in neural computations, supporting fundamentally different modes of cortical operation based on transient dynamics (Buonomano and Maass, 2009) or predictive processing (Rao and Ballard, 1999; Keller and Mrsic-Flogel, 2018).

More generally, we call any information processing mode *state-dependent* if the output of a circuit at time *t* depends not only on the input at that time, but also on previous values of the input and of the output itself. Experimental evidence points to the widespread existence of state-dependent processing and of circuit features that can support it in visual cortex. For instance, neural coding of visual stimuli depends on the behavioral context (Niell and Stryker, 2008; Khan and Hofer, 2018), highlighting the existence of feedback connections projecting from other parts of the brain; cortico-cortical or cortico-thalamic feedback is also compatible with experimental observations (Lamme et al., 1998; Issa et al., 2018; Marques et al., 2018). The effects of short-term adaptation can be seen in the reduction of the responses to repeated stimuli or in those to continuous versus transient stimuli, two phenomena that may increase in intensity along the ventral stream (Grill-Spector et al., 2006; Kohn, 2007; Kaliukhovich et al., 2013; Webster, 2015; Stigliani et al., 2019; Fritsche et al., 2020). It is interesting to note that these ideas are now also starting to influence the design of artificial systems, e.g., recent deep neural network architectures that extend classic convolutional networks with the addition of recurrent and adaptive elements (Tang et al., 2018; Kar et al., 2019; Kreiman and Serre, 2020; van Bergen and Kriegeskorte, 2020; Vinken et al., 2020).

**Figure 5:**
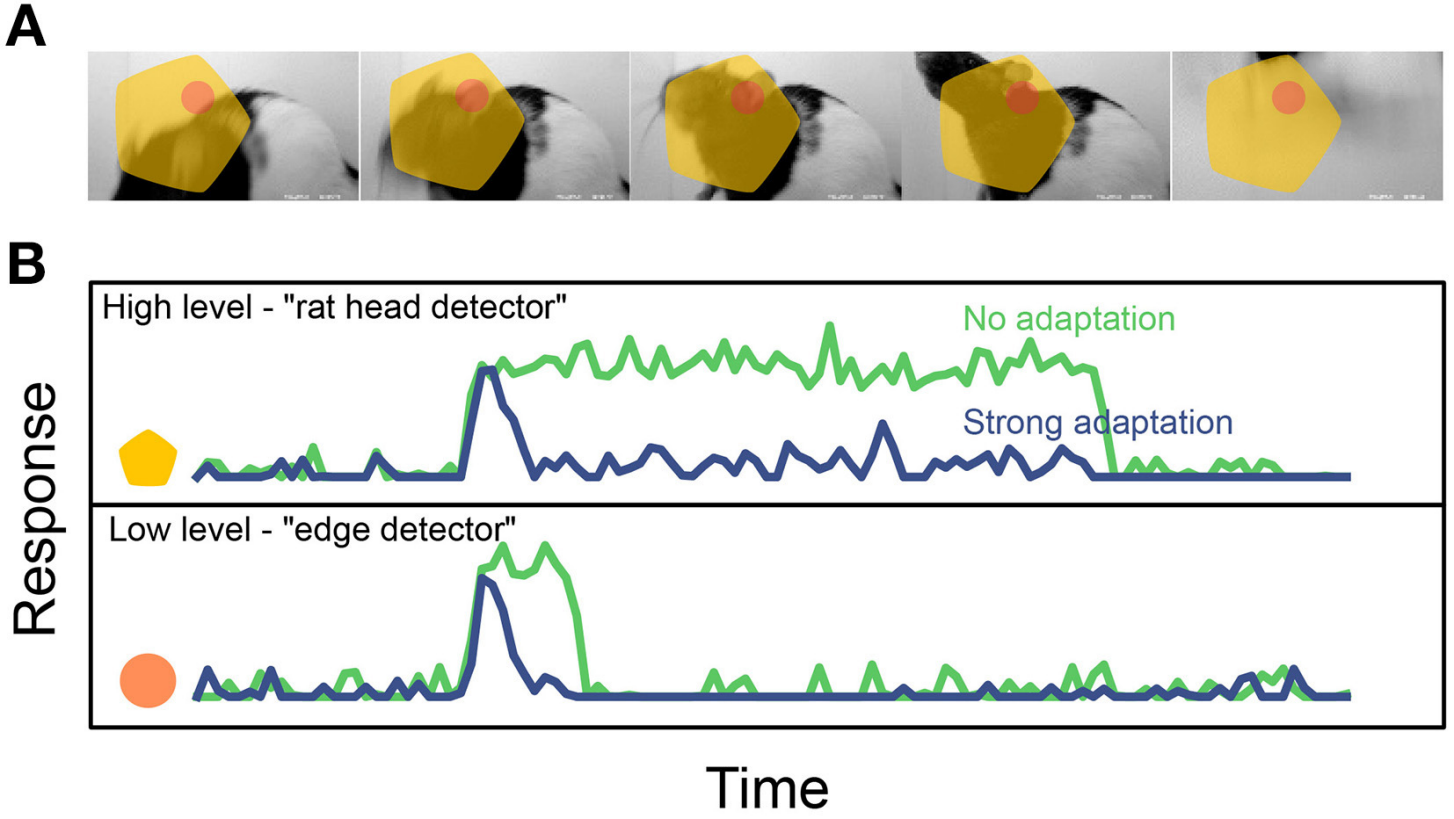
Effect of short-term adaptation on the response timescale of high- vs. low- level feature detectors (cartoon). **(A)** Dynamical visual stimulus (movie frames). Orange dot, yellow shape: idealized receptive field of a low-level feature detector neuron (“edge detector,” orange) and a high-level feature detector (“rat head detector,” yellow). **(B)** Single-trial response of the two example neurons, when adaptation is absent (green trace) and when adaptation is strong (blue trace). Note how adaptation shortens the timescale of the response. Reproduced from Piasini et al. (2021). Activity traces in B are obtained by simulating a simple neural encoding model, also described in details in Piasini et al. (2021).

Whatever their functional roles, state-dependent mechanisms can have dramatic effects on the temporal dynamics of neural codes. For instance, imagine a visual stimulus containing an object undergoing identity-preserving transformations, such as the rat moving its head in Figure 5. In absence of state-dependent processing, a neuron that is selective for the presence of a rat head will fire continuously as long as the rat head is within the field of view. On the other hand a different neuron, selective for a low-level image feature such as the presence of an oriented edge within a small region, would only fire briefly whenever the correct stimulus enters its receptive field. As discussed above, this should result in a *slower* encoding for the high-level feature than for the lower-level one (Figure 5B, green). However, if state-dependence is now added in the form of a simple short-term adaptation mechanism, the difference in timescale between these hypothetical neurons can be dramatically reduced, as both units switch to encoding feature onset/offset rather than feature presence (Figure 5B, blue). In this simplified example, there is a tension between metabolic and functional efficiency: adaptation decreases energy consumption by reducing the total activity (using a crude form of predictive coding, where the prediction at each point in time is that the stimulus will persist in the current state), but makes it harder for the system to build invariant representations of the stimuli, which are advantageous on functional grounds.

Another way in which state-dependent processing can affect the timescale of neural codes is by acting on *intrinsic* timescales. Intrinsic timescales describe the temporal extent over which fluctuations around the average response to a given stimulus are correlated (whenever it is necessary to distinguish them from the timescales of a stimulus' neural representation, we will call the latter *response* timescales). Operationally, multiple definitions of intrinsic timescales are possible, but for concreteness we will use the following Piasini et al. (2021). If $X_{t}={\left\{x_{k}^{\left(t\right)}\right\}}_{k=1}^{K}$ is the activity of one neuron at time *t* recorded over *K* identical repetitions of the experiment (trials), the *intrinsic correlation* at time lag Δ is the average correlation coefficient between the activity at time *X*_*t*_ and *X*_*t*+Δ_:


$$C\left(\Delta \right)=\frac{1}{T-\Delta }\overset{T-\Delta }{\underset{t=1}{\sum }}\frac{\mathrm{Cov}\left[X_{t},X_{t+\Delta }\right]}{\sqrt{\mathrm{Var}\left[X_{t}\right]\mathrm{Var}\left[X_{t+\Delta }\right]}},$$

where *T* is the duration of the recording, such that 1 ≤ *t* ≤ *T*. The intrinsic timescale is then a measure of the characteristic time over which *C* decays as Δ grows. In this sense, the intrinsic timescale captures the temporal range over which “temporal noise correlations” are present. Intrinsic timescales are thought to increase along cortical hierarchies (Murray et al., 2014; Runyan et al., 2017; Wang, 2022), possibly reflecting an increase in the importance of certain classes of state-dependent processes, such as temporal integration or more complex dynamics emerging from recurrent connectivity (Chaudhuri et al., 2015; Piasini et al., 2021).

### 4.3. Response and intrinsic timescales increase along the visual cortical pathway

**Figure 6:**
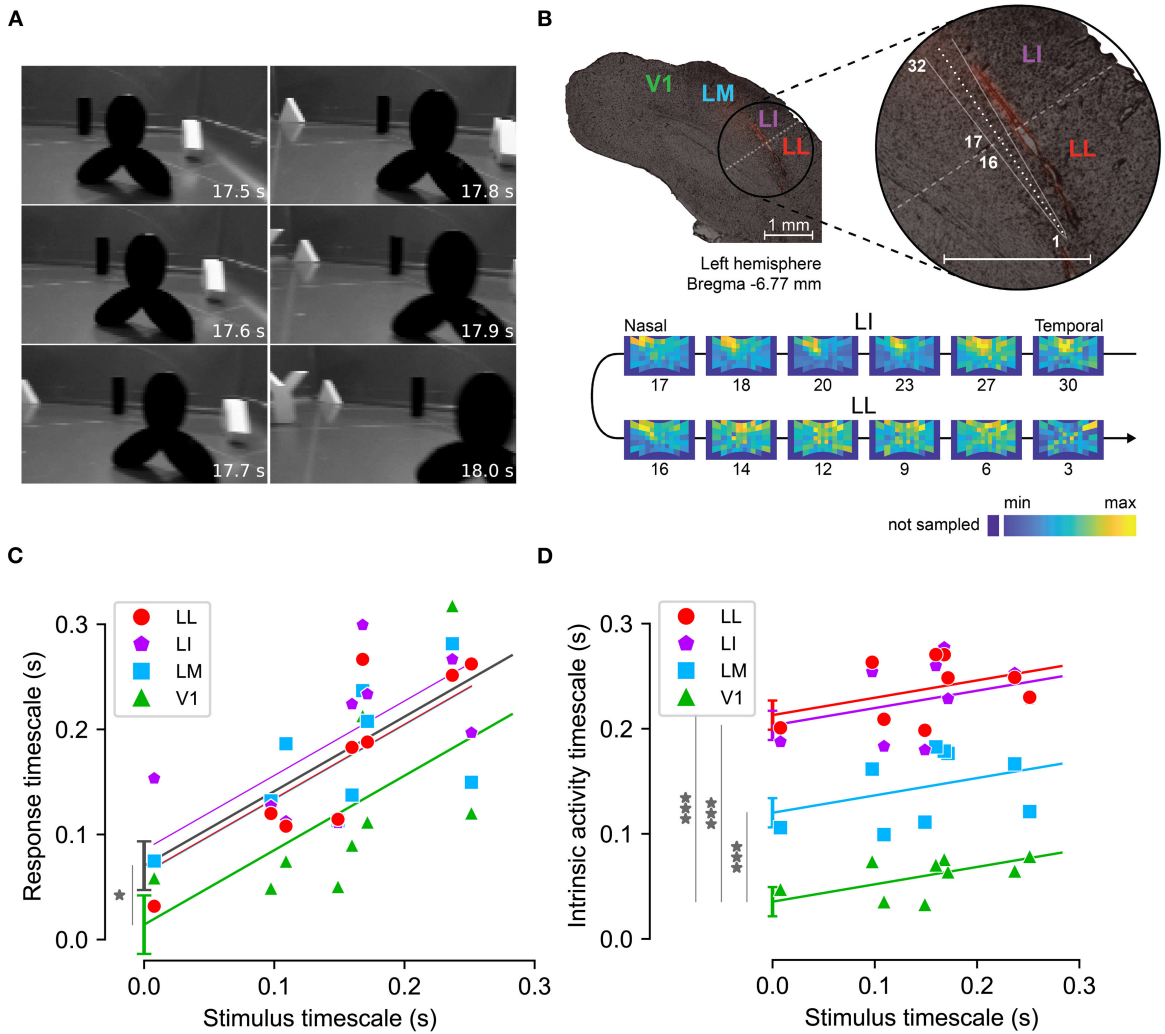
Measuring response and intrinsic timescales along the rat analog of the ventral visual stream. **(A)** Example frames from one of the nine movies used as visual stimuli. **(B)** Functional identification of rat cortical areas. Top: example slice of rat visual cortex, obtained from one of the rats where recordings were performed. Red fluorescence indicates the insertion path of the multielectrode silicon probe, schematized in white. Bottom: firing intensity maps showing the RFs of the units recorded at selected recording sites along the probe (indicated by the numbers under the RF maps). The reversal in the progression of the retinotopy between sites 16 and 17 marks the boundary between areas LI and LL (shown by a dashed line on the top panel). **(C)** Response timescales (y-axis) measured across the cortical hierarchy for stimuli with different timescales (x-axis). Markers indicate empirical estimates, lines indicate linear regression with common slope across areas and varying intercept. Gray line indicates a regression where all extrastriate areas (LM, LI, LL) are pooled together and compared to V1. **(D)** Same as **(C)**, for intrinsic timescales. Adapted from Piasini et al. (2021).

While the classic view of invariant representations in the ventral stream suggests a straightforward increase of neural timescales along the hierarchy, the discussion above highlights that the existence of state-dependent mechanisms implies a more complex picture. To gain some insight into this matter, Piasini et al. (2021) performed an empirical study of the timescales of visual cortical representations of dynamic stimuli (Figure 6).

Piasini et al. (2021) recorded the activity elicited by the presentation of dynamic visual stimuli (movies) in four areas of rat visual cortex, which form the rodent analog of the visual ventral stream (Vermaercke et al., 2014; Glickfeld and Olsen, 2017; Tafazoli et al., 2017; Vinken et al., 2017; Kaliukhovich and Op de Beeck, 2018). Analysis of the data showed that response timescales depended strongly on the timescale of the stimulus, and were significantly larger in extrastriate cortex than in V1 (Figure 6C). This suggests that adaptation and other state-dependent processing mechanisms do not prevent an increase of slowness of the neural code along the hierarchy, a correlate of increasing invariance to identity-preserving transformations. Moreover, analysis of intrinsic timescales revealed a weaker dependence on the timescale of the stimuli (as expected), but a very strong increase along the cortical hierarchy, compatibly with previous results in monkey (Murray et al., 2014) and in behaving mice (Runyan et al., 2017) suggesting that the importance of state-dependent and adaptive processes is also increasing along the cortical hierarchy. These results were confirmed also by re-analysing previously published data recorded in mouse (Siegle et al., 2021) and in awake rat (Vinken et al., 2016).

## 5. Challenges for the future

We would not expect the efficient coding hypothesis to hold throughout the brain in the elementary form of maximizing information transmission. One fundamental reason for this is that there are statistical and resource limitations. Even if we just consider texture encoding, note that as the complexity of the textures under consideration increases, the dimensionality of the corresponding texture space quickly grows. This leads, on the one hand, to difficulty in sampling the relevant natural-image ensemble: even if two texture dimensions differ in terms of their statistics in natural scenes, the number of samples required to characterize the difference may simply be too large for any realistic organism to achieve the required adaptation. Moreover, even if we solved the statistical-sampling problem, the actual improvements in information transfer obtained by adapting to very high-dimensional texture spaces might not be worth the brain circuitry required to perform the required encoding. Put differently, efficient coding might be limited by logistics. It is of course possible in principle to include resource or sampling limitations as additional constraints in our optimization problem. This would ensure that the efficient coding solution we find obeys those limitations. In practice, however, we might not have a quantitative understanding of some of these limitations. In this case the best we can do is employ the most accurate model we have, but keep in mind that its predictions will be affected by the incomplete set of constraints used in the model.

Apart from logistics, another factor in potential conflict with efficient coding is the fact that animals encode information not with the end goal of information transmission, but in order to perform behaviors that are helpful for their survival. While in many cases, efficiently encoding information will be in alignment with this objective, in other cases different considerations might be more important. For instance, a very efficient encoding that is extremely hard to decode (or turn into helpful behavior) might not be very useful. We therefore see another important way in which we expect simplistic information-optimizing efficient coding to only be part of the story: organisms need to fulfill other roles apart from encoding information. Indeed, it would be useful to develop a formalism which combines coding efficiency, computational realizability and effectiveness, and, critically, behavioral goals in a more complete normative theory of neural circuit organization.

Despite these caveats, all sensory systems must adapt in some way to the statistics of their natural inputs in order to perform well. Below we describe three promising directions for the future.

### 5.1. Explaining shape sensitivity from object shape distributions

The success of the efficient coding approach to texture processing in the brain suggests another question: can we explain the distribution of cells responsive to different kinds of shapes in the ventral visual pathway? Studies have shown that individual cells in V4 are responsive to fragments with different shapes and curvatures (Pasupathy and Connor, 1999, 2001, 2002). Likewise cells in IT (and its rodent analog) are selective for specific objects, again with different numbers of edges, corners, and curvatures (Tanaka, 1996; Hung et al., 2005; Rust and DiCarlo, 2010). The degree of invariance to image transformations also increases with depth in the visual pathway (Riesenhuber and Poggio, 1999; DiCarlo and Cox, 2007; Rust and DiCarlo, 2010; DiCarlo et al., 2012; Tafazoli et al., 2017). Perhaps the statistics of these responses are adapted to the distribution of shapes and shape fragments in natural scenes. Studies have demonstrated distinctive shape and curvature statistics in natural images, and have connected these statistics to visual perception (Geisler et al., 2001; Geisler and Perry, 2009). It would be interesting to directly relate these sorts of visual scene statistics to the neural circuits in the ventral visual pathway.

### 5.2. Explaining cell type distributions in parallel information pathways

Another outstanding challenge is to explain the structure of the parallel pathways that appear in many parts of the brain, where an information stream is processed by multiple cell types, each selective for part of the stream, and then transmitted through parallel fibers in a nerve tract to other parts of the brain (Perge et al., 2012). It is possible that efficient coding can provide a theory of such decompositions (Balasubramanian, 2015). The retina in particular is a tempting target for such an analysis as it presents a classic feedforward neural network architecture that winnows down the vast amount of data in the incident photons into an Ethernet cable's worth of information transmitted by about 20 parallel ganglion cell channels (Koch et al., 2006; Perge et al., 2009; Balasubramanian, 2015). To take such an approach, it will likely be important to include constraints associated with the *decoder*—i.e., the region of the brain that must use limited computational resources to rapidly read the information encoded in the incident parallel pathway. Indeed, recent work (Gjorgjieva et al., 2019) suggests that functional diversity in sensory neurons can be understood by balancing the mutual information between stimuli and responses against the error incurred by computationally constrained decoders. It would be interesting to understand if the repertoire of nonlinear feature detectors in the retina (Gollisch and Meister, 2010) can be understood in this way.

### 5.3. Methods based on machine learning complementing more normative approaches

Finally, the advent of deep learning may provide an interesting new approach to understanding the logic of neural circuits deeper in the brain, where the guiding principle is that circuits beyond the sensory periphery must self-organize through local learning rules to achieve whatever tasks are behaviorally necessary. Indeed, some authors have suggested that the hierarchy of visual cortical areas should be understood in analogy with the layers of a deep network (Yamins et al., 2014; Yamins and DiCarlo, 2016; Schrimpf et al., 2020; Muratore et al., 2022; Nayebi et al., 2022). Of course these circuits ultimately operate on inputs drawn from the natural world, and hence should adapt through learning to both scene statistics and the target task. This approach has shed light on the presence of grid cells in the entorhinal cortex (Banino et al., 2018; Cueva and Wei, 2018; Sorscher et al., 2019; Cueva et al., 2020) and on the repertoire of retinal ganglion cells (McIntosh et al., 2016). This perspective also raises the intriguing possibility that circuits in the brain are not just organized to encode information efficiently, but also to learn efficiently (Teşileanu et al., 2017).

## Author contributions

TT, EP, and VB conceived of and performed the research described in this paper. All authors contributed to the conception of the article and the writing. All authors contributed to the article and approved the submitted version.

## Funding

This work was supported in part by NSF grant PHY-1734030, NIH grant R01EB026945, and NSF grant CISE 2212519.

## Conflict of interest

The authors declare that the research was conducted in the absence of any commercial or financial relationships that could be construed as a potential conflict of interest.

## Publisher's note

All claims expressed in this article are solely those of the authors and do not necessarily represent those of their affiliated organizations, or those of the publisher, the editors and the reviewers. Any product that may be evaluated in this article, or claim that may be made by its manufacturer, is not guaranteed or endorsed by the publisher.

## Footnotes

^1^If patches are chosen randomly, the assumption of constant texture will sometimes be violated. This should however be relatively infrequent if the image patches are not too large.

^2^We call a certain multi-point correlation informative if knowing its value significantly reduces our uncertainty about the underlying luminance pattern *in the natural image ensemble that we are considering*.

^3^Note that the naming of texture group 2 differs between Victor and Conte (1991) and Tkačik et al. (2010): group 2 in the former refers to group 3 in the latter. Group 2 from Victor and Conte (1991) is not used in Tkačik et al. (2010).
